# Influence of Cognitive Impairment on the Recovery of Subjects with Subacute Stroke Undergoing Upper Limb Robotic Rehabilitation

**DOI:** 10.3390/brainsci11050587

**Published:** 2021-04-30

**Authors:** Irene Aprile, Giulia Guardati, Valeria Cipollini, Dionysia Papadopoulou, Serena Monteleone, Alessandra Redolfi, Romina Garattini, Gianluigi Sacella, Fulvia Noro, Silvia Galeri, Maria Chiara Carrozza, Marco Germanotta

**Affiliations:** 1 IRCCS Fondazione Don Carlo Gnocchi ONLUS, 50143 Florence, Italy; iaprile@dongnocchi.it (I.A.); giulia.guardati@gmail.com (G.G.); vcipollini@dongnocchi.it (V.C.); dpapadopoulou@dongnocchi.it (D.P.); 2 IRCCS Fondazione Don Carlo Gnocchi ONLUS, 20121 Milan, Italy; smonteleone@dongnocchi.it (S.M.); aredolfi@dongnocchi.it (A.R.); rgarattini@dongnocchi.it (R.G.); gsacella@dongnocchi.it (G.S.); fnoro@dongnocchi.it (F.N.); sgaleri@dongnocchi.it (S.G.); mccarrozza@dongnocchi.it (M.C.C.); 3 The Biorobotics Institute, Scuola Superiore Sant’Anna, 56127 Pisa, Italy

**Keywords:** rehabilitation, robotics, stroke, executive function, attention, memory

## Abstract

Cognitive decline is often present in stroke survivors, with a significant impact on motor recovery. However, how specific cognitive domains could impact motor recovery after robotic rehabilitation in patients with stroke is still not well understood. In this study, we analyzed the relationship between cognitive impairment and the outcome of a robot-mediated upper limb rehabilitation intervention in a sample of 51 subacute stroke patients. Participants were enrolled and treated with a set of robotic and sensor-based devices. Before the intervention, patients underwent a cognitive assessment by means of the Oxford Cognitive Screen. To assess the effect of the 30-session rehabilitation intervention, patients were assessed twice with the following outcome measures: the Fugl-Meyer Assessment for Upper Extremity (FMA-UE), to evaluate motor function; the Upper limb Motricity Index (MI), to evaluate upper limb muscle strength; the Modified Barthel Index (mBI), to evaluate activities of daily living and mobility. We found that deficits in spatial attention and executive functions impacted the mBI improvement, while language, number processing, and spatial attention deficits reduced the gains in the FMA-UE. These results suggest the importance to evaluate the cognitive functions using an adequate tool in patients with stroke undergoing a robotic rehabilitation intervention.

## 1. Introduction

Robotic therapy is a well-established approach for upper limb rehabilitation after stroke, to increase the amount and intensity of the therapy and to standardize the treatment [1]. Recently, the importance of robotics to personalize the treatment, based on the patient’s performance, has been underlined. Indeed, it permits to modulate the characteristics of the training, in terms of movement required, strength assistance, and explored workspace. A systematic review [2] showed that electromechanical and robot-assisted arm training improved activities of daily living in people after stroke, as well as the function and the muscle strength of the affected arm. Moreover, two recent studies on large samples showed that it is at least as effective as conventional therapy [3,4].

Besides motor impairment, cognitive decline is often present in stroke survivors. Several studies confirmed the high prevalence of cognitive impairment after stroke [5,6,7,8,9,10] and underlined its significant influence on motor learning strategies [11], functional recovery, and quality of life.

However, the impact of cognitive decline on the clinical outcome of a robotic intervention is scarcely considered; therefore, few data on the topic are available. A recent systematic review [12] on 66 articles and 2214 participants highlighted that only 10 over 66 trials (15%) enrolled stroke participants with impaired cognition, whereas 50 (76%) excluded those with impaired cognition. Finally, the remaining 6 trials did not clearly report if patients with a cognitive decline were included or not. Therefore, this review confirmed the scarcity of information on the impact of cognitive impairment on the outcome in patients with stroke undergoing robotic rehabilitation. Only a recent paper showed that cognitive impairment is a negative predictor of functional and motor outcomes in patients with stroke after upper limb robotic therapy [13].

We hypothesize that cognitive decline deeply impacts motor recovery after robotic rehabilitation in patients with stroke. The aim of this paper is to evaluate if the impairment of the cognitive functions influences the recovery of the upper limb motor performance in patients after stroke undergoing robotic rehabilitation.

## 2. Materials and Methods

### 2.1. Study Design and Participants

This is a secondary analysis of a longitudinal study [14] approved by the institutional Ethics and Experimental Research Committee on 13 March 2019 (FDG_13.3.2019) and registered on Clinicaltrial.gov (ClinicalTrials.gov Identifier: NCT04164381). We recruited consecutive subjects with (a) one ischemic or hemorrhagic stroke (verified by MRI or CT); (b) age between 35 and 85 years; (c) a time since the stroke onset within 6 months; (d) cognitive abilities adequate to understand the experiments and follow the instructions (Token test ≥ 26.5, corrected by age and school level) and (e) with upper limb impairment (Fugl-Meyer Assessment score ≤ 58). We excluded patients with: (a) history of recurrent stroke; (b) behavioral and cognitive disorders and/or reduced compliance that could interfere with the treatment; (c) fixed contraction in the affected limb (ankylosis, Modified Ashworth Scale equal to 4), and (d) severe deficits in visual acuity. The study was conducted following the International Conference on Harmonization Good Clinical practice guidelines and the Declaration of Helsinki. All participants gave written informed consent before study participation.

### 2.2. Assessment

At baseline, demographic and clinical data were recorded; Moreover, patients underwent a cognitive screening: specifically, we used the Italian version of the Oxford Cognitive Screen (OCS), recently developed with the specific aim to describe the cognitive deficits after stroke [15]. The scale consists of 10 tasks encompassing five cognitive domains: attention and executive function, language, memory, number processing, and praxis. Furthermore, it includes a brief evaluation of visual field defects. We used the Italian version of the scale [16], where the cut-offs for each domain, corrected by age, sex, and education levels, are reported.

To assess the effect of the rehabilitation intervention, patients were assessed before and after robotic rehabilitation with the following outcome measures: the Fugl-Meyer Assessment for Upper Extremity (FMA-UE) [17], to evaluate motor function; the Upper limb Motricity Index (MI) [18], to evaluate upper limb muscle strength; the Modified Barthel Index (mBI) [19], to evaluate activities of daily living and mobility.

### 2.3. Treatment

**Figure 1:**
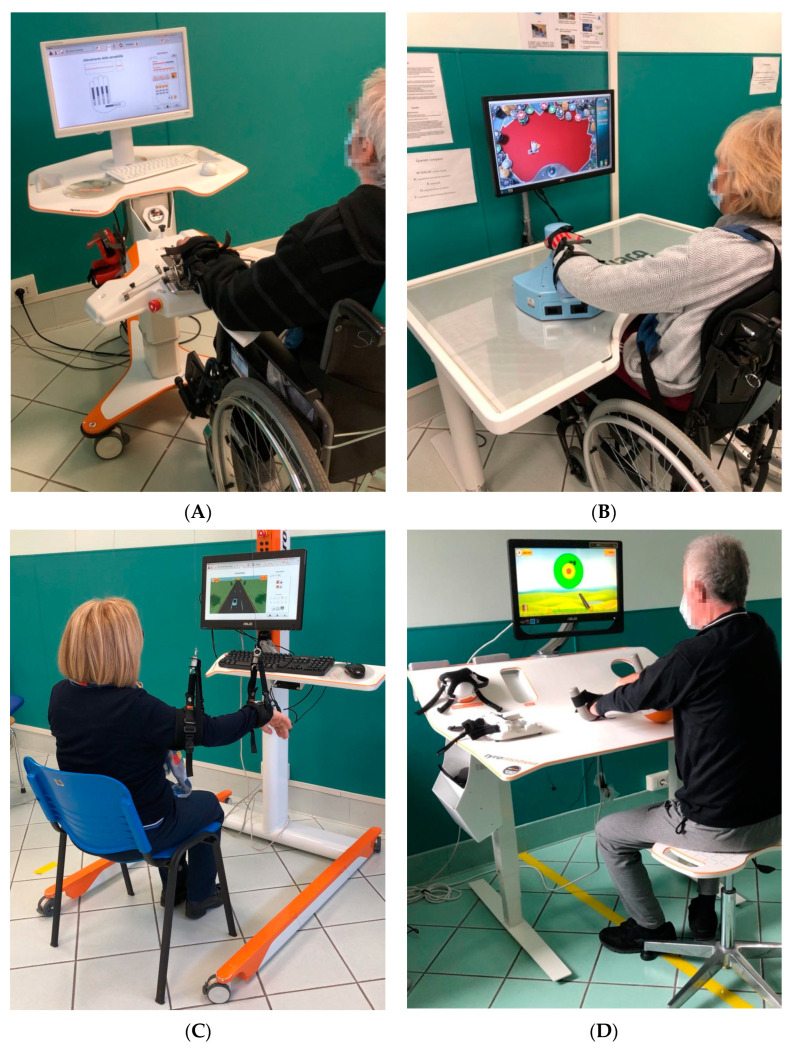
Robotic and sensor-based devices used to treat the upper limb: (**A**) Amadeo (Tyromotion); (**B**) Motore (Humanware); (**C**) Diego (Tyromotion); and (**D**) Pablo (Tyromotion).

Patients were treated with a set of robotic and sensor-based devices showed in Figure 1 (Motore, from Humanware, Pisa, Italy; and Amadeo, Diego, and Pablo, from Tyromotion, Graz, Austria) [20,21]. Motore is a robotic device that allows passive, active, and active-assistive planar movements of the shoulder and elbow joints; Amadeo is a robotic device that allows passive, active, and active-assistive finger flexion and extension movements; Pablo is a sensor-based system that allows unsupported three-dimensional movements of the shoulder, elbow, and wrist joint, both unimanual and bimanual; Diego is a robotic system that allows three-dimensional, unimanual and bimanual, movements of the shoulder joint, with arm weight support. During the treatment, patients performed both motor and cognitive tasks, and the devices provided visual and auditory feedback to help them.

The upper limb treatment was performed daily for 45 min, 5 days a week, for 30 sessions. More details on the treatment are reported elsewhere [14]. Further, patients underwent a comprehensive rehabilitation program including individual conventional physiotherapy (six times/week), lasting 45 min, focused on lower limbs, sitting and standing training, balance, and walking. Patients with language disorders performed speech training.

### 2.4. Statistical Analysis

Visual inspection and the Shapiro–Wilk test showed that data did not meet the criteria for parametric analysis, and therefore, non-parametric tests were used.

The percentages of patients with cognitive impairment, according to the OCS scores, were summarized for the whole sample, and patients with a left or a right hemiparesis, separately. The percentage of patients with cognitive impairments were compared according to the affected side, by means of the Fisher Exact test.

To investigate the impact of cognitive deficits on the recovery of upper limb motor performance, strength, and activities of daily living, the change from baseline of the clinical scales (the FMA-UE, the MI, and the mBI) were compared between patients with or without cognitive deficits, according to the OCS domains, using the Mann-Whitney U test. When significant, the Cohen’s d was computed to assess the effect size (small, d = 0.2; medium, d = 0.5; and large, d = 0.8). For all the statistical analyses, a *p*-value of 0.05 was deemed significant. Statistical analysis was performed with SPSS (IBM SPSS Statistics for Windows, Version 25.0, Armonk, NY, USA).

## 3. Results

### 3.1. Baseline Assessment

The demographic and clinical characteristics of the sample are given in Table 1. Regarding activities of daily living dependence, the sample showed a severe disability (measured using the mBI) associated with a moderate/severe upper limb impairment and strength (measured using the FMA-UE and MI, respectively).

Table 2 showed the percentages of patients obtaining a pathological score in the Oxford Cognitive Screen at baseline. In particular, the highest percentage of the pathological score was detected in number processing (*calculation* task, about 70%); spatial attention (*hearts cancelation* task, about 60%); language, memory, and executive functions (*sentence reading*, *episodic memory*, and *baseline score* tasks respectively, about 50%). Lower percentages were found in praxis (*imitation task*, about 25%) and perception (*visual field* task, only one case). Comparing patients with right and left hemiparesis, a statistically significant difference were detected in *sentence reading*, *recall and recognition*, and *number writing* tasks (with a higher prevalence in patients with right hemiparesis) and *object asymmetry* task (with a higher prevalence in patients with left hemiparesis).

### 3.2. Rehabilitation Outcomes

**Figure 2:**
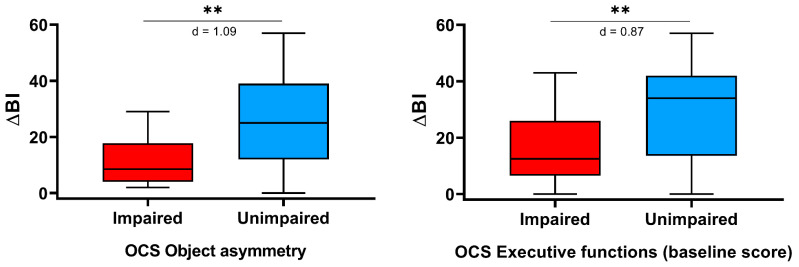
Oxford Cognitive Screen (OCS) scores impacting on the recovery in the activity of daily living, as measured by the change from baseline of modified Barthel Index (ΔBI). The asterisks indicate a statistically significant difference (** *p* < 0.01). Finally, Cohen’s d is reported.

**Figure 3:**
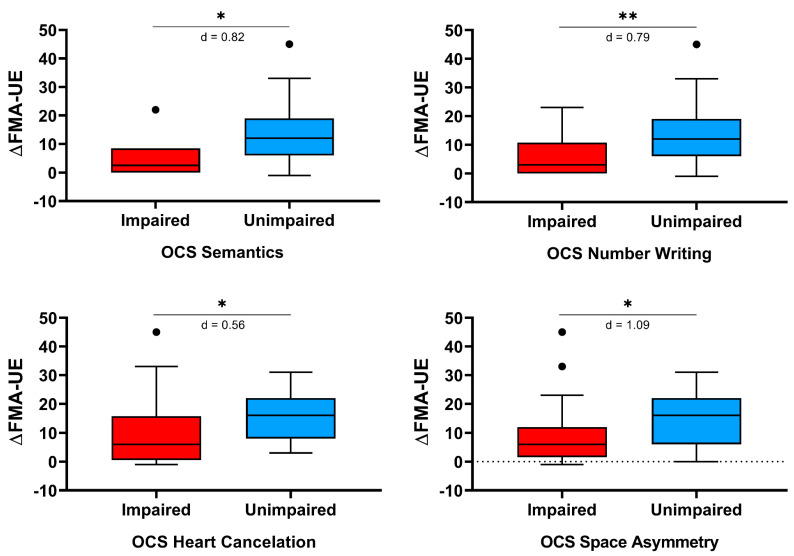
Oxford Cognitive Screen (OCS) scores impacting motor recovery, as measured by the change from baseline of Fugl-Meyer Assessment for the Upper limb (ΔFMA-UE). The asterisks indicate a statistically significant difference (* *p* < 0.05; ** *p* < 0.01). Finally, Cohen’s d is reported.

Table 3 reports the changes from baseline of the considered clinical scales, for patients with or without cognitive impairment in the domains assessed by the OCS. The improvement in motor function, as measured by the FMA-UE, was statistically significantly lower in patients showing cognitive deficits in the following OCS tasks: s*emantics*, *number writing*, *heart cancelation*, and *space asymmetry*. Similarly, the improvement in the activities of daily living, as assessed by the mBI, was lower in patients showing a pathological score in the *object asymmetry* and the *executive function* (*baseline score)* tasks. On the contrary, the improvement in motor strength, as measured by the MI, did not differ between patients with or without a pathological score in the OCS tests. As showed in Figure 2 and Figure 3, the effect size was medium for one score (*heart cancellation* task, change in the FMA-UE) and strong for the remaining five tasks.

## 4. Discussion

Cognitive impairment is considered a priority in the rehabilitation path of patients after stroke [22] because it influences the recovery of motor function and ability in life daily activities. Cognitive functions, like attention or memory, have a crucial role during motor and functional rehabilitation programs; in fact, high attention and memory may enable people to engage better with the exercises with a high ability to cope with the proposed tasks [23]. Indeed, the limited transfer of upper limb motor improvement to other tasks or activities of daily living [24] could be due to coexistent cognitive impairment. Then, both cognitive and motor functions should be evaluated and treated in patients after stroke during the rehabilitation treatment, because the former can influence the recovery of the latter.

In this context, the robotic treatment has been proposed as a viable approach to improve motor performance and activities of daily living; however, the same attention was not paid to the cognitive area. Our results showed that the cognitive status can impact the gain in motor and activities of daily living performance after a robotic rehabilitation intervention. To the best of our knowledge, only one study focused on the influence of cognitive status on motor recovery after robotic upper limb rehabilitation in stroke patients [13]. The authors’ findings are in line with our results since they suggest that the initial cognitive functions are positively associated with the functional outcome after robot-assisted therapy. However, it is worthy to note that the cognitive decline of patients was measured using the Mini-Mental State Examination and, therefore, the authors did not provide information about the cognitive functions that could impact the most on the rehabilitation outcome.

To better understand our results it is important to describe the clinical, motor, and cognitive function of our sample. About half of the sample was affected by right hemisphere damage, while half was affected by left hemisphere damage; moreover, as expected, ischemic stroke was prevalent. Regarding the activities of daily living dependence and the motor performance of the upper limb, our sample showed a severe disability, associated with a moderate to severe impairment in upper limb motor function and strength.

With regard to the assessment of cognitive functions, we used, as a screening tool, the Oxford Cognitive Screening. It is important to note that our group was part of the Italian OCS Group and participated in the study detecting cognitive impairment in stroke patients using the OCS [16] and, therefore, our researchers were adequately trained to administer this tool.

Similar to the results of the paper of Mancuso et al. [16], in our sample left-hemisphere damage patients had a significantly higher frequency of impairments in the language (*sentence reading* task) and in the memory (*recall and recognition* task) domains. In the *hearts cancellation* task, a higher percentage of right-hemisphere damage patients showed inattention for left space (64.3%), but also a higher percentage of left-hemisphere damage patients showed a higher percentage of right inattention for the right space (60.9%). On the contrary, we detected a higher percentage of failure in the number domain (43.5%) in left-hemisphere damage patients than right-hemisphere damaged patients (14.3%); moreover, left-hemisphere damage patients had a significantly worse performance in the *object asymmetry* task of the spatial attention (32.1%) than left-hemisphere damaged patients (4.3%). This means that more than 50% of our sample showed an impairment of the attention (measured by *heart cancellation* task), independently of the side of the lesion. In addition, half of the patients with left-hemisphere damage and about 30% of the patients with right-hemisphere damage showed an impairment of the spatial exploration, while a significantly high percentage of patients with left-hemisphere damage showed a severe deficit seeing the object in its left side. Likely the differences between our and Mancuso’s sample are due to the difference in the time since the stroke onset, higher in Mancuso’s sample than in ours.

The main result of our study was that the impairment in specific cognitive functions, evaluated using a specific tool, negatively influences the post-stroke recovery of the upper limb motor performance and ability in activities of daily living after upper limb robotic rehabilitation. In particular, the improvement in patients with language impairment (semantic functions and abilities to understand and write the numbers heard) was significantly lower than in unimpaired patients. This result can be due to the difficulty of the patient with language impairment to correctly understand the proposed exercises and tasks during upper limb robotic rehabilitation. As expected, patients with an impairment in attention and visuospatial functions showed a lower improvement of the upper limb performance than patients in which these cognitive functions were spared. Similarly, the impairment of visual-spatial functions and executive functions negatively influenced the improvement in the ability of the daily living activities.

These results were identified since the OCS was able to separately assess memory, language, number cognition, praxis, executive functions, and attention. Moreover, it is important to note that the administration of OCS is compatible with the presence of severe language impairments, as it includes items that do not require language-based answers (e.g., the patient has to indicate the answer among different visual alternatives); similarly, the influence of left unilateral spatial neglect is minimized by arranging targets vertically, whenever appropriate.

Our result underlined that a cognitive evaluation is important to better address the rehabilitation treatment since the cognitive impairment can negatively influence the upper limb motor performance; however, it is important to highlight that cognitive impairment (as visuospatial functions, attention, and executive functions) in patients after stroke can improve after robotic upper limb rehabilitation. Indeed, as shown in our previous study, upper limb robotic rehabilitation can improve cognitive function as attention and visual-spatial function [14].

In conclusion, this study confirmed our hypothesis, since in our sample we found a relationship between cognitive impairment and post-stroke upper limb motor recovery. In addition, this study confirmed the importance to evaluate cognitive functions in patients with stroke undergoing rehabilitation, using an adequate and specific tool. On the basis of our data, we believe that new robotic exercises, enriched by visual and acoustic feedback, virtual reality application, and cognitive goals, and, therefore, able to combine motor and cognitive training, could enhance the outcome of the treatment.

## Figures and Tables

**Table 1:** Demographic and clinical characteristics of the sample (N = 51).

Entry Characteristics	Mean (SD) or N (%)
Age	68.4 (12.4)
Sex (men/women)	29 (56.9%)/22 (43.1%)
Dominant side (Right/Left)	47 (92.2%)/4 (7.8%)
Education years	10.2 (3.8)
Stroke type (Ischemic/Hemorrhagic)	36 (70.6%)/15 (29.4%)
Affected side (Right/Left)	23 (45.1%)/28 (54.9%)
Days from index stroke to enrollment	74.6 (41.3)
Language impairment	11 (21.6%)
Neglect syndrome	10 (19.6%)
Fugl-Meyer Assessment for Upper Extremity	21.5 (18.1)
Motricity Index	37.3 (27.9)
Modified Barthel Index	40.3 (18.3)

**Table 2:** Number and percentage of patients who failed the Oxford Cognitive Screen tests (N = 51). Values in bold indicate a statistically significant difference, according to the Fisher Exact test (*p* < 0.05).

Domain	Task	Whole Group		Right Hemiparesis		Left Hemiparesis		*p*
		N	%	N	%	N	%	
Language	Picture naming	20	39.2%	12	52.2%	8	28.6%	0.149
	Semantics	6	11.8%	4	17.4%	2	7.1%	0.390
	Sentence reading	24	47.1%	15	65.2%	9	32.1%	**0.026**
Memory	Orientation	13	25.5%	7	30.4%	6	21.4%	0.529
	Recall and recognition	21	41.2%	14	60.9%	7	25.0%	**0.012**
	Episodic memory	28	54.9%	14	60.9%	14	50.0%	0.573
Number	Number writing	14	27.5%	10	43.5%	4	14.3%	**0.029**
	Calculation	36	70.6%	19	82.6%	17	60.7%	0.125
Perception	Visual Field	1	2.0%	0	0.0%	1	3.6%	>0.999
Spatial attention	Hearts cancelation	32	62.7%	14	60.9%	18	64.3%	>0.999
	Space asymmetry	22	43.1%	8	34.8%	14	50.0%	0.395
	Object asymmetry	10	19.6%	1	4.3%	9	32.1%	**0.015**
Praxis	Imitation	13	25.5%	7	30.4%	6	21.4%	0.529
Executive function	Baseline score	26	51.0%	11	47.8%	15	53.6%	0.781
	Shifting score	14	27.5%	6	26.1%	8	28.6%	>0.999

**Table 3:** Comparison of the change from baseline of the modified Barthel Index (ΔBI), the Motricity Index (ΔMI), and the Fugl-Meyer Assessment for Upper extremity (ΔFMA-UE) between patients with or without impaired cognitive functions, according to the Oxford Cognitive Screen. Values in bold indicate a statistically significant difference, according to the Mann-Whitney U test (*p* < 0.05).

Domain			ΔBI		ΔMI		ΔFMA-UE	
			Mean (SD)	*p*	Mean (SD)	*p*	Mean (SD)	*p*
**Language**	Picture Naming	impaired	21.3 (15.1)	0.678	12.5 (11.6)	0.112	8.5 (7.5)	0.053
		unimpaired	23.5 (15.9)		18.5 (13.3)		14.2 (11.0)	
	Semantics	impaired	15.3 (8.1)	0.268	13.3 (8.6)	0.875	**5.2 (8.4)**	**0.037**
		unimpaired	23.6 (16.0)		16.5 (13.4)		**12.8 (10.0)**	
	Sentence Reading	impaired	22.0 (14.5)	0.872	14.0 (13.1)	0.201	9.5 (9.4)	0.074
		unimpaired	23.1 (16.6)		18.1 (12.6)		14.1 (10.3)	
**Memory**	Orientation	impaired	21.2 (14.3)	0.863	14.1 (13.1)	0.428	8.2 (8.4)	0.14
		unimpaired	23.1 (16.0)		16.9 (12.9)		13.2 (10.4)	
	Recall and Recognition	impaired	24.7 (13.5)	0.329	15.5 (12.8)	0.81	11.0 (8.8)	0.715
		unimpaired	21.1 (16.8)		16.6 (13.2)		12.6 (11.0)	
	Episodic Memory	impaired	21.3 (14.1)	0.576	17.5 (14.1)	0.615	11.7 (10.8)	0.655
		unimpaired	24.2 (17.2)		14.5 (11.3)		12.2 (9.5)	
**Number processing**	Number Writing	impaired	19.4 (11.8)	0.428	11.6 (11.1)	0.133	**6.6 (8.8)**	**0.007**
		unimpaired	23.8 (16.6)		17.9 (13.2)		**14.0 (9.9)**	
	Calculation	impaired	21.4 (14.3)	0.521	14.6 (12.9)	0.198	11.1 (10.9)	0.158
		unimpaired	25.3 (18.2)		20.0 (12.6)		13.9 (7.8)	
**Perception**	Visual Field	impaired	26.0 (0.0)	0.784	9.0 (0.0)	0.588	3.0 (0.0)	0.431
		unimpaired	22.5 (15.6)		16.3 (13.0)		12.1 (10.1)	
**Spatial attention**	Hearts Cancelation	impaired	19.0 (14.0)	0.054	15.1 (12.9)	0.423	**10.0 (10.9)**	**0.015**
		unimpaired	28.6 (16.2)		18.0 (13.0)		**15.2 (7.7)**	
	Object Asymmetry	impaired	**11.4 (9.3)**	**0.01**	12.5 (15.4)	0.263	9.7 (13.5)	0.171
		unimpaired	**25.3 (15.5)**		17.0 (12.3)		12.5 (9.2)	
	Space Asymmetry	impaired	21.8 (14.3)	0.909	15.8 (13.5)	0.886	**9.0 (11.4)**	**0.011**
		unimpaired	23.2 (16.5)		16.4 (12.7)		**14.2 (8.5)**	
***Praxis***	Imitation	impaired	18.2 (14.6)	0.289	15.7 (13.8)	0.828	11.5 (12.5)	0.573
		unimpaired	24.1 (15.6)		16.3 (12.7)		12.1 (9.3)	
**Executive function**	Baseline Score	impaired	**16.5 (12.5)**	**0.006**	15.6 (13.0)	0.872	10.7 (11.4)	0.135
		unimpaired	**28.9 (15.9)**		16.7 (13.0)		13.3 (8.6)	
	Shifting Score	impaired	27.9 (12.5)	0.078	17.8 (14.0)	0.567	14.2 (9.4)	0.200
		unimpaired	20.6 (16.1)		15.5 (12.6)		11.1 (10.3)	

## Data Availability

The data presented in this study are available on request from the corresponding author.
